# The Outcome of Status Epilepticus and Long-Term Follow-Up

**DOI:** 10.3389/fneur.2019.00427

**Published:** 2019-04-26

**Authors:** László Horváth, István Fekete, Márk Molnár, Réka Válóczy, Sándor Márton, Klára Fekete

**Affiliations:** ^1^Department of Pharmaceutical Surveillance and Economy, Faculty of Pharmacy, University of Debrecen, Debrecen, Hungary; ^2^Department of Neurology, Faculty of Medicine, University of Debrecen, Debrecen, Hungary; ^3^Faculty of Art, Institute of Political Science and Sociology, University of Debrecen, Debrecen, Hungary

**Keywords:** status epilepticus, antiepileptic drug treatment, outcome, risk factors, follow-up

## Abstract

**Objective:** This study was to investigate the outcome of status epilepticus (SE) associated with antiepileptic therapy during SE and in follow-up period, risk factors including age, co-morbidities, pre-existing epilepsy, and etiology in the East-Hungarian region.

**Methods:** A prospective cross-sectional database was compiled from outpatient files between 2013 and 2017. Follow-up ended on 30.06.2018.

**Results:** One hundred and thirty five episodes (male: 68, 50.4%) were evaluated, mean age and follow-up time being 64.1 ± 13.9 years and 39.9 ± 14.2 months, respectively. Of the 89 patients with pre-existing epilepsy, 34 failed to visit the outpatient unit regularly. Case fatality rate was 25.2% and 31 patients (30.7%) died after discharge due to co-morbidities; their mean survival time was 10.44 ± 8 months. Focal, generalized and combined type epilepsies were diagnosed in 67 patients (49.6%), 47 patients (34.8%), and 21 patients (15.6%) of SE, respectively. Nine patients had non-convulsive SE (NCSE). Mean seizure-free period was 6.8 ± 6.9 months. Patients taking carbamazepine (20.9%; OR: 0.37, 95%CI: 0.16–0.82; *p* = 0.018), levetiracetam (27.5%; OR: 0.51, 95%CI: 0.27–0.97; *p* = 0.041), or valproate (11.1%; OR: 0.18, 95%CI: 0.05–0.61; *p* = 0.0043) were expected to achieve seizure freedom after SE. The worst outcome was linked to advanced age, etiology, new onset status epilepticus, NCSE, and focal status epilepsy.

**Conclusion:** This study highlights the importance of regular care and patient follow-up.

## Introduction

Status epilepticus (SE) is a condition and most extreme form of epilepsy (1), which leads to abnormal and prolonged seizure (at least 5 min). In case SE persists over 30 min, it may have severe long-term consequences (2). Referring to the new classification scheme of SE, there are two operational dimensions of the definition: time point 1 (T1) is associated with abnormally prolonged seizure, when therapy should be initiated, while time point 2 (T2) is related to the time of on-going seizure activity involving a risk of long-term consequences (2).

SE is one of the most common neurological emergencies (1). It is a potentially life-threatening situation which needs a prompt and particular treatment (3) in order to prevent cerebral damage due to initial excitotoxicity (4). Treatment is urgent because GABA sensitivity decreases and the sensitivity to excitotoxic neurotransmitters increases rapidly, leaving only a short time interval for effective treatment. SE may also have life-time consequences and, especially in refractory SE, the probability of becoming epileptic is higher (5).

Cases of reported refractory and super-refractory SE (SRSE) are uncommon but very important clinical problems due to treatment difficulties, consequences, and high case fatality (1, 2, 4). Nevertheless, they probably occur more often than thought, especially if one thinks of non-convulsive SE. Five to ten percent of refractory SE patients turn out to have super refractory SE (6).

The incidence of SE falls in the range of 10–41/100,000 (1). Despite the newer generation of antiepileptic drugs (AEDs), the management of epilepsy and SE has not been resolved yet (7).

In this study, the patients' age and co-morbidities influenced the outcome of SE (1). Common etiologies included non-compliance, stroke, metabolic disorders, and alcohol withdrawal among adult patients. Of the people with epilepsy, 15% had SE during their lifetime; however, pre-existing epilepsy went undiscovered in more than 50% of SE patients.

In the current study, we focused on the outcome (short and long-term mortality and seizure freedom) of SE in view of antiepileptic therapy during SE and in the follow-up period, risk factors such as age, co-morbidity, pre-existing epilepsy, as well as the underlying pathology, in the East-Hungarian region.

## Methods

Data were retrieved from the patients' files, covering the period between 01.01.2013 and 31.12.2017 in a retrospective view. The subjects had been treated for SE at the neurointensive unit of a tertiary teaching hospital and coded with status epilepticus diagnoses in accordance with the International Classification of Diseases by the World Health Organization (8). Each admission of the same patient was considered as a single case. In this study, the patients were followed-up until 30.06.2018. The department provides care only for adult patients. The catchment area is ~548,000 inhabitants.

In every case, data collection included issues as follows: age, gender, cause, history of previous epilepsy, former intracranial surgery, treatment with antiepileptic drugs before, during and after SE, and other medicines regularly taken for CNS, MRI scan, EEG, and comorbidities. The 21 EEG electrodes were placed according to the International 10–20 system, and digital recording was used. All EEGs were evaluated by a board-certified clinical neurophysiologist. In the case of convulsive SE, EEG was done to classify the type of the seizure (focal, or generalized). According to the recommendations, and if it was clinically reasonable (e.g., impairment of consciousness), a post-SE EEG was done in order to exclude a transition to NCSE and to monitor the effectiveness of the therapy. NCSE was diagnosed according to Beniczky et al. (9). Depending on the therapy and response to it, EEG was repeated 24–48 h later. Unfortunately, continuous EEG monitoring was not available at the time of the study.

The following age groups were considered for pooling: 18–39, 40–64, 65–80, and over 81-years-old.

Regarding the status epilepticus, the following data were collected: type of seizure (focal, secondary generalized, generalized, non-convulsive, and unknown seizures according to the ILAE definition (10, 11), antiepileptic treatment, besides the benzodiazepine used primary and in case of survival, used for maintenance therapy.

Notable co-morbidities were as follows: diabetes mellitus, hypertension, hypercholesterolemia, renal failure, liver failure, heart failure, cancer, Parkinson's disease, stroke etc.

New onset status epilepticus (NOSE) was considered if the patient did not have known seizures previously.

Refractory SE (RSE) was diagnosed if the patients did not respond to standard SE treatment, i.e., receiving adequate doses of initial benzodiazepine followed by a second AED (12). In case of SRSE, the definition of Shorvon et Ferlisi was used, i.e. SE could not be terminated or it recurred within 24 h; also, when propofol or midazolam were necessary, including cases in which SE recurred on the reduction or withdrawal of these drugs (4).

### Statistical Analysis

Statistical analysis was carried out using the SPSS for Windows 19.0 (SPSS Inc. Chicago, USA) and Microsoft Office Excel 2007. Beside the basic statistics, two-sample *T*-test, and Fisher's exact test were used to analyse our patients' data. Categorical variables were assessed using Pearson χ^2^ test. Odds ratios were calculated. Significant differences were considered if *p* < 0.05.

Ethical approval was obtained from the Regional and Institutional Ethics Committee (DE KK RKEB/IKEB: 5037-2018).

## Results

### Basic Characteristics

The diagnosis of SE was established in 121 patients (male: 61; 50.4%). As eight patients (6.6%; male 3, female: 5) had had two or more admissions due to SE, a total of 135 episodes (male: 68, 50.4%) were evaluated. The patients' mean age was 64.1 ± 13.9 years.

Based on the distribution by age, 87 (71.9%) of the patients were between 40 and 80 years old. Among them, the working/active age group included 50 (41.3%) patients.

The mean follow-up time was 39.9 ± 14.2 months. The longest follow-up was 66 months and the shortest was 7.5 months.

The prevalence of SE was in the range of 9.4–14.7/100,000 inhabitants/year.

The seasonality of prevalence showed the highest occurrence in December and January followed by August.

According to the classification of Trinka et al. (2), previous epilepsy was known in 89 patients (73.6%), the mean age being 62.2 ± 14.4 years. Of the 32 patients with unknown previous epilepsy (26.4%; mean age: 70.1 ± 12.35 years), 22 died during hospitalization, all could be classified as symptomatic SE. Among the survivors (10 patients), there was only one patient who had no symptomatic abnormality in the background. So only this one could fit the definition of the ILAE epilepsy diagnosis. One third of the patients did not visit epilepsy outpatient units regularly. Of the 89 patients with known epilepsy, 37.1% had focal seizures without secondary generalization and 23.6% had focal seizures with secondary generalization. Among the patients with generalized epilepsy (39.3%) seizure types were as follows: generalized tonic-clonic, myoclonic and absence, i.e., 33, 1 and 1 cases, respectively. Only one patient had a history of Lennox-Gastaut syndrome. Altogether, 91% had symptomatic epilepsy among patients with a history of this disease.

Among all the patients with SE, 78 and 23 patients had RSE and SRSE, respectively. New onset refractory status epilepticus (NORSE) was observed in 18 patients among those having refractory SE. The number of NOSE and NORSE cases with undetermined causes were so few (3 and 2 patients, respectively) that far-reaching conclusion cannot be drawn.

All of the SE patients had hypertension.

### Etiology

Unknown etiology of SE was revealed in nine cases but in the majority of the patients, several causes were identified. In the different age groups, the particular causes showed varying pictures in terms of etiology (Table 1).

**Table 1:** Distribution of causes in different age groups.

**Etiology**	**18–39 years-old *N* = 15 (A/B)**	**40–64 years-old *N* = 44 (A/B)**	**65–80 years-old *N* = 51 (A/B)**	**≥81 years-old *N* = 25 (A/B)**
Tumor; primary CNS tumor	0	8;5 (5/3)	9;5 (6/3)	1;1 (1/0)
Systemic infection	4 (4/0)	9 (7/2)	13 (9/4)	13 (6/7)
Meningitis, encephalitis	2 (2/0)	1 (0/1)	1 (1/0)	0
Alcohol	2 (2/0)	17 (12/5)	5 (4/1)	2 (2/0)
Congenital abnormality	7 (6/1)	2 (2/0)	0	0
Metabolic disorder	0	2 (2/0)	4 (4/0)	3 (3/0)
Non-compliance	0	11 (11/0)	5 (5/0)	2 (2/0)
Stroke; ischemic	0	10;8 (6/1)	14;5 (12/2)	8;8 (8/0)
Intracranial traumatic bleeding	0	2 (2/0)	1 (0/1)	0
Sleep deprivation	2 (2/0)	0	0	0
Unknown	1 (1/0)	1 (0/1)	5 (5/0)	2 (0/2)

*(A patient may have multiple etiologies; the data are discussed from the point of view of SE)*.

*N: number of patients*.

*(A/B): number of patients with pre-existing epilepsy/ number of new onset epilepsy cases*.

**Figure 1:**
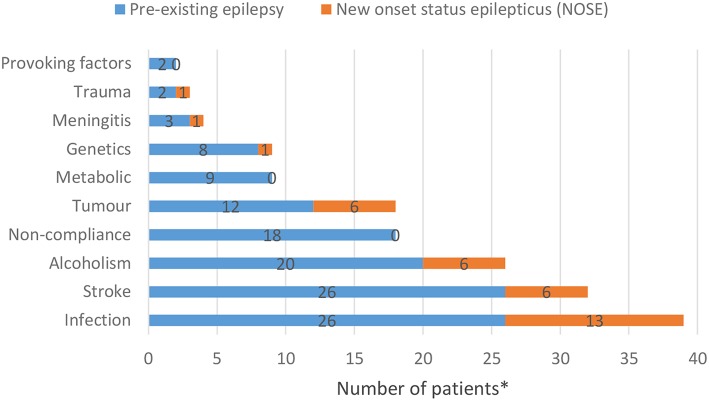
Most common causes of status epilepticus among people with a history of epilepsy and NOSE patients (^*^A patient may have multiple etiologies; the data are discussed from the point of view of SE).

Among patients with previously known epileptic seizures, the most common causes of status epilepticus were infections, stroke, alcoholism and non-compliance (Figure 1) while among patients with NOSE, infections, alcoholism, stroke and tumor were at the top of the list (Figure 1). Only two-thirds of the people with epilepsy were regularly supervised by an epileptologist prior to SE; these patients all had refractory epilepsy.

Just under 50% of cases were focal, and focal epilepsy with secondary generalization (41 patients; 30.4%; 26 patients; 19.3% respectively); generalized epilepsy was diagnosed in 34.8% (47) of patients and combined-type epilepsies amounted to 15.6% (21 patients) of status epilepticus. Nine patients were diagnosed with non-convulsive status epilepticus (NCSE). Of them, three patients had generalized seizure and four patients focal epilepsy with secondary generalization prior to NCSE.

### Antiepileptic Drug Treatment

All known people with epilepsy took one, two, and three or more types of AEDs, 34 (52.3%), 21 (32.3%), 12 (18.5%), respectively. Interestingly, if we compare the pattern of AED use among SE patients with a history of epilepsy with the data of people with epilepsy in our outpatient care unit (published earlier) (13), the differences are significant (*p* = 0.0014) (34 [52.3%] vs. 894 [69.7%]; 21 [32.3%] vs. 286 [22.3%]; 12 [18.5%] vs. 102 [8%]).

In order to control seizures, one, two, and three or more AEDs were used in 15 patients (11.1%); 41 patients (30.4%); and 79 patients (58.5%); respectively, subsequent to the first line benzodiazepine (diazepam or clonazepam) during SE. No significant difference was found between the number of AEDs taken before SE and the AEDs administered during SE.

General anesthesia was necessary in 23 (17%) patients. Midazolam, ketamine and propofol were administered in 16 (11.9%), 4 (3%), and 9 (6.7%) cases, respectively. Seven of the patients had generalized type of epilepsy, eleven patients had focal epilepsy with or without secondary generalization (7, 4 respectively), four patients suffered from a combined type and one patient had NCSE.

Certain older generation AEDs such as valproate, clonazepam, phenytoine, and carbamazepine (supp.) as well as newer type ones including levetiracetam, and lacosamid were used in the treatment of SE. Even if a nasogastric tube had to be inserted treatment with AEDs such as oxcarbazepine and lamotrigine were continued.

***Among the patients who survived SE*** (101 patients), 85.1% (86 individuals) took one or two AEDs (49 and 37 patients, respectively) at discharge to maintain seizure freedom. Only 15 patients were discharged with three or more AEDs to take. The number of AEDs per patient was 1.7 ± 0.7. The number of AEDs is significantly (<0.0001) higher if the results of patients surviving SE and our previous findings (1.4 ± 0.56) of people with epilepsy at the outpatient department are compared (14).

The prescriptions of newer type AEDs were significantly higher at discharge than at admission (*p* < 0.005), but the number of older type AEDs showed a variety as well (Table 2). Among our patients, the ones taking carbamazepine, levetiracetam and/or valproate had the highest probability to achieve seizure freedom. The choice of AED on discharge had no significant effect on mortality (Table 2). The need for general anesthesia was independent of the add-on administration of levetiracetam and/or lacosamide in SRSE.

**Table 2:** Number of prescribed AEDs on discharge and the outcome as of 30 June 2018.

**AED**	**Number of prescriptions**	**Number of seizure free patients**	**Odds ratio (95% CI; *p*-value)**	**Number of deaths**	**Odds ratio (95% CI; *p*-value)**
Levetiracetam	69	19	0.51 (0.27–0.97; 0.041)^*^	25	0.95 (0.51–1.76; 0.88)
Carbamazepine	43	9	0.37 (0.16–0.82; 0.018)^*^	18	1.31 (0.65–2.63; 0.47)
Valproate	27	3	0.18 (0.05–0.61; 0.0043)^*^	8	0.68 (0.28–1.65; 0.52)
Oxcarbazepine	13	1	0.13 (0.02–1.01; 0.033)	4	0.74 (0.22–2.5; 0.77)
Lamotrigine	14	5	0.94 (0.3–2.93; 1)	3	0.44 (0.12–1.63; 0.26)
Lacosamide	16	3	1.1 (0.25–4.83; 1)	8	1.73 (0.62–4.82) 0.42
Clonazepam	6	2	0.85 (0.15–4.75; 1)	3	1.73 (0.34–8.85; 0.67)
Clobazam	4	0	N/C	1	0.56 (0.06–5.5; 1)
Phenytoin	2	0	N/C	2	N/C
Rufinamide	2	0	N/C	0	N/C
Primidone	1	0	N/C	1	N/C
Topiramate	1	1	N/C	1	N/C
Zonisamide	1	1	N/C	1	N/C

*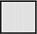, Older type AEDs. N/C not computable*.

“*” represent statistically significant values.

### Outcome

The discharged patients' mean ***survival time*** was 10.44 ± 8 months. Seventy patients have survived SE (mean age: 55.8 ± 14.6 years) and 25 of them achieved seizure freedom. The mean seizure free period was 6.8 ± 6.9 months (the shortest seizure free time was 1 day and the longest one was 5 years).

Sixty-five patients (53.7%) ***died*** during the period investigated, primarily due to co-morbidities especially common in the advanced age group. Case fatality rate was 25.2% (Table 3) among all examined SE patients 22 of which suffered from NOSE. The death rate among NOSE patients was significantly higher than the mortality among previously people with epilepsy (*p* = 0.009).

**Table 3:** Death and survival of patients with status epilepticus.

	**Death ratio**			**Survival**
	**All**	**During hospitalization**	**After discharge**	
Number of patients	65	34	31	70
Mean age ± SD (year)	72.7 ± 9.9	75.4 ± 9.3 (ref.)	**68.7** **±** 10.7^*^	**55.8** **±** 14.6^*^
Male	29	17	12	39
Female	36	17	19	31
Mortality (%)	48.1	25.2	23	N/A

*N/A not applicable*.

* *p ≤ 0.05*.

Young patients had a much better chance to survive (Table 3). There was no difference between male and female mortality rates. If the time course of death was examined, in-hospital case fatality was the highest and a second peak was detected at 6 months. Mortality increased by age (*p* < 0.0001). Apparently, younger patients died after discharge.

**Figure 2:**
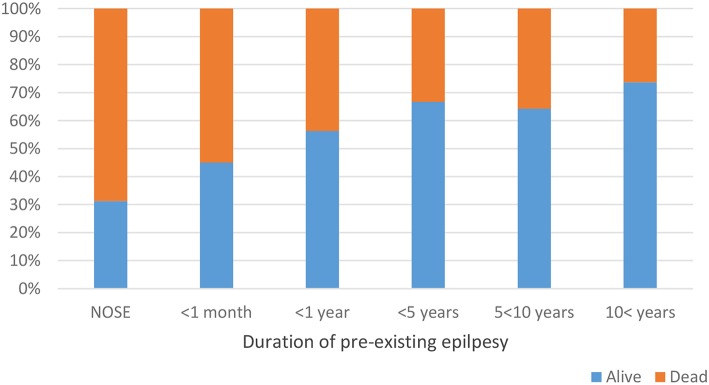
Duration of pre-existing epilepsy and status epilepticus mortality. Only one in nine NCSE patients survived (*p* = 0.014).

Among patients with pre-existing epilepsy, the highest mortality was observed in case the duration of epilepsy was < 1 month (Figure 2).

The leading causes of SE among deceased patients were stroke (42.86%), tumor (33.33%) and infection (23.81%). Mortality within a co-morbidity group was due to tumor (28%), stroke (25.71%), and infection (20.83%). The probability of death increased significantly (*p* = 0.0021) with the number of co-morbidities (Table 4). All patients who had had cancer died.

**Table 4:** Outcome by etiology.

**Etiology**	**Dead**	**Alive**	**Odds ratio**	**95% Confidence Interval**		***p*-value**
				**Lower limit**	**Upper limit**	
Tumor (CNS)	17^*^(6)	**1 (1)**	**25.78**	**3.34**	**198.88**	**<0.0001**
Systemic infection	23	16	2.02	0.98	4.19	0.067
Alcohol	9	17	0.59	0.25	1.41	0.29
Meningitis, encephalitis	1	3	0.39	0.04	3.83	0.63
Congenital abnormality	1	8	0.15	0.018	1.2	0.08
Metabolic disorder	5	4	1.37	0.35	5.3	0.74
Non-compliance	5	13	0.42	0.14	1.24	0.14
Stroke (ischaemic)	13 (1)	19 (13)	0.78	0.36	1.7	0.56
Intracranial traumatic bleeding	1	2	0.59	0.053	6.66	1
Unknown	2	7	0.32	0.07	1.6	0.18

*(A patient may have multiple etiologies; the data are discussed from the point of view of SE)*.

* *p ≤ 0.05*.

Most of the deceased patients had focal epilepsy with or without secondary generalization (61.5%), 21.5% of patients had generalized seizure (Table 5). NCSE was observed in 12.3% of the SE patients. By the types of SE, NCSE showed the highest mortality, followed by focal epilepsy (59.7%) and generalized epilepsy (31.1%).

**Table 5:** Mortality by seizure type.

**Seizure type**	**Dead**	**Alive**	**Odds ratio (95% CI; *p*-value)**
Generalized	15	31^*^	0.38 (0.18–0.8; 0.0075)^*^
Focal	39^*^	27	2.39 (1.2–4.77; 0.01)^*^
Focal without sec. gen.	25^*^	15	2.29 (1.07–4.89; 0.024)^*^
Focal with secondary generalization	14	12	1.33 (0.56–3.13; 0.33)
^b^Combined	10	11	0.98 (0.38–2.48; 1)
NCSE at onset	1	1	1.08 (0.07–17.6; 1)
All patients with NCSE^a^	8^*^	1	9.86 (1.18–79.4; 0.014)^*^

* *p ≤ 0.05*.

a *This group contained all patients who had not only NCSE*.

b *Combined: during treatment several types of seizure originating from different foci*.

The number of AEDs given to cease seizures were inconsistent with mortality.

Using older and/or newer type of AEDs (administered as second or third line drugs) did not influence the odds of death during the status epilepticus.

The AEDs used during SE did not influence mortality significantly. General anesthesia did not influence survival and seizure freedom significantly.

Co-administered other drugs belonging to the ATC N05 group (47 patients) before SE did not influence the seizure freedom after SE, but the mortality among patients taking at least one drug was favorable (OR: 0.41 95%CI: 0.1969–0.8571; *p* = 0.02). They took chlordiazepoxide, alprazolam, antidepressants, etc.

## Discussion

### Basic Characteristics

SE, also known as status epilepticus, is an important and life-threatening form of epilepsy. This study is the first in our region to summarize SE cases and evaluate the treatment and outcome.

Just as in other SE studies, the gender ratio was nearly equal (15) and more than half of the patients were elderly. Our research is of special importance since almost half of the patients were still active, i.e., they belonged to the working-age population. Among middle-aged patients, the most common etiologies included alcohol consumption, non-compliance and stroke. Meanwhile, infection and stroke most often affected elderly patients. The onset of stroke was typical at an older age but in our cohort—similarly to alcoholism—the incidence of stroke was high in younger patients.

No strict seasonality was observed but data aggregation by month showed peaks in January and December during the winter, and in August during the summer. There was no close relation between SE and holidays either. Although this is an interesting finding, we could not find unequivocal clarification for it. However, it must be noted, that stable and unstable atmospheric pressure and temperature go from one extreme to the other in Hungary in the aforementioned months. Motta et al. (16) also reported increased seizure frequency in unstable weather conditions.

A quarter of the patients had NOSE. They were older than those with previously diagnosed epilepsy, moreover, they usually suffered from severe co-morbidities. Their history of epilepsy started with SE and mortality among them was higher. Despite the adequate therapy of SE, their prognosis was poor. Based on our results, previous epilepsy was 74.4%, quite similar to those of a Norwegian study (73%) (15), but lower (43%) than in a large prospective cohort study (17). It should be noted that the latter publication only examined refractory or super refractory SE cases. On the basis of our findings, one third of the patients diagnosed with epilepsy did not visit their epileptologist on a regular basis. This is regarded to be as an example of non-compliance/adherence behavior because, in this country, yearly follow-up by an epileptologist is necessary to subsidize AED prescriptions. So, we think that this type of non-adherence may contribute to the evaluation of SE. This emphasizes the importance of care in preventing SE. Regular care is of similar importance as, for example, preventing SUDEP (sudden unexpected death in epilepsy) (18).

### Etiology

Infection was the leading cause of SE in our cohort. Among the patients with pre-existing epilepsy, non-compliance, alcoholism and stroke followed infections by frequency, while alcoholism, stroke and tumor were the most common etiologies among NOSE patients. In a Norwegian SE cohort, cerebrovascular diseases, intracranial tumors, low AED levels, and neurodegenerative disorders (15) were listed as the most common cause for SE.

Alcohol- and stroke-related cases of SE were less common in our study than in an article by Leppik (19). He found stroke to have caused 52.3% of the cases of SE among the elderly adults and 17.7% in middle-aged adults, while, in our study, the figures were 16.3 and 7.4%, respectively. It is worth mentioning that stroke was remarkable in younger age groups and among the 65–80 year-old patients. As far as stroke patients are regarded, ischaemic stroke occurred more frequently in all age groups, except for the patients aged between 65 and 80 years; the latter group was more often hit by haemorrhagic stroke. The high occurrence of haemorrhagic stroke in SE is interesting, because ischaemic stroke is more frequent in the elderly. All SE patients had a history of hypertension, which is one of the most important risk factors of stroke.

In a recently published study, Ulvin et al. found the level of generalized convulsive SE at 67% in the non-refractory SE group, and at 47% in refractory SE (15). In contrast with Ulvin et al. in our study, the focal form of SE was most common, just as in the publications by Sutter et al. (20) and Novy et al. (21). Possible contradictions among these findings may be due to disparities in etiology.

### Treatment

The number of AEDs prior to status epilepticus did not influence the number of AEDs used to control SE. Compared to other SE studies, fewer patients needed general anesthesia (17 vs. 41% by Ulvin; and 9.8% by Delaj). The choice of the AED did not influence the need for general anesthesia. Some medications (e.g., benzodiazepines) used in SE may have severe adverse drug reactions like respiratory depression, so for NCSE patients (e.g., stroke) a good alternative therapy might be newer type of AED (22).

Most of the patients needed only one or two AEDs at discharge, and newer types of AEDs were in favor: we assumed that the role of drug interactions among multimorbid patients was an important argument.

Seizure freedom was significantly longer among patients taking levetiracetam, carbamazepine, and valproate.

### Outcome

In accordance with the literature, mortality from SE was high (19) and age dependent.

In-hospital mortality was 25%, in SE studies showing high variability in different studies (9–37%) (17, 20, 21, 23). However, the mortality in pre-existing epilepsy was less than in NOSE in our study. According to our findings, NOSE could be considered as a high risk factor for mortality (Figure 2) emphasizing the importance of NOSE.

Etiology also had a considerable impact on the outcome beyond SE. All patients who had cancer died.

In focal SE mortality was significantly higher than in the generalized type. Focal neurological lesions such as stroke and tumors caused mostly focal epileptic seizures, which may explain the outcome (Table 5).

The number and type of older and newer AEDs did not influence the outcome of SE. In terms of seizure remission, there were no significant differences between new and old type AEDs; important was to terminate SE (24). In his publication, Schmidt came to a similar conclusion concerning refractory epilepsy: newer types of AEDs were not more efficient than the old ones (22). SE might also be regarded as an extreme refractory type of convulsion.

Only one out of nine patients survived NCSE. All of these patients had severe co-morbidities and were of advanced age. In general, the low occurrence of NCSE might be due to being underdiagnosed in non-neurological wards, where the patients were treated for their primary diseases on one hand, on the other hand in lack of long-term EEG on the intensive care unit and Stroke Unit ward less patients are discovered with NCSE. Implementing long-term EEG on these wards in case of predictors (large infarct size, large atherothrombotic etiology, high NIHSS score on admission) would help the diagnosis of NCSE and lead to early treatment and better outcome (25).

We are aware that our study has several limitations. First of all, the current study is an observational study and not a randomized, controlled trial. Therefore, selection bias might have affected the results. Secondly, treatment options and definitions have changed during the investigated period. Unfortunately, because the several aetiological and triggering effects only low case numbers would have been achieved with subgroup analysis. Nevertheless, this study has invaluable assets including prospective data collection and detailed information on all subjects. Further strength of our study may be the real-life data sets leading to the better understanding of real-life clinical settings and the outcome of routine status epilepticus treatment.

In summary, several conditions complicate the picture in everyday practice so real-life data are essential in order to understand real patients in the ward. No strict seasonality of SE cases was observed. The worst outcome of SE was linked to advanced age, etiology, new onset status epilepticus (NOSE), NCSE and focal status epilepsy. The choice of the AED did not influence the need for general anesthesia. The administration of newer type AEDs in the SE treatment may have an impact on the prescription pattern after discharge, however older type AEDs are a reasonable choice to achieve seizure freedom after SE. Seizure freedom was significantly higher among patients taking levetiracetam, carbamazepine and valproate. This study highlights the importance of regular care and follow-up of patients.

## Ethics Statement

According to the institutional and national guidelines for retrospective analysis of encrypted data without intervention the Regional Ethical Committee allowance is needed: DE KK RKEB/IKEB: 5037-2018.

## Author Contributions

KF, LH, and IF led the initiative and revised the drafted document. KF, LH, MM, and RV selected abstract, extracted data, and drafted the manuscript. SM, LH, KF, and RV is involved in investigation, data curation, data analysis, and writing the original draft. IF is involved in supervision. All authors are involved in the conceptualization, methodology, review, and editing. All authors approved the final version.

### Conflict of Interest Statement

The authors declare that the research was conducted in the absence of any commercial or financial relationships that could be construed as a potential conflict of interest.
